# Successful Management of Uterine Prolapse in Pregnancy: A Case Report of Avoiding a Cervical Laceration by the Reduction of Massive Edema

**DOI:** 10.7759/cureus.60456

**Published:** 2024-05-16

**Authors:** Daisuke Tachibana, Akihiro Hamuro, Kohei Kitada, Takuya Misugi

**Affiliations:** 1 Obstetrics and Gynecology, Osaka Metropolitan University Graduate School of Medicine, Osaka, JPN

**Keywords:** cervical laceration, cervical edema, delivery, pregnancy, uterine prolapse

## Abstract

We report a case of uterine prolapse in pregnancy, which was successfully managed before delivery. A 35-year-old woman (G2P1) complained consistently of a protruding uterus at 36 weeks gestation, and an engorged uterine cervix without tenderness, urinary disturbance, and incontinence were recognized (Pelvic Organ Prolapse Quantification (POP-Q) score C: +7). Manual retraction of the edematous cervix was gently performed with gauze packing, and strikingly improved edema of the cervix with a POP-Q score of C: -2 was observed one day after the gauze packing. Induction of labor was planned due to a suspected large-for-gestational-age infant, and the patient uneventfully delivered at 39 weeks gestation without any obstacles to delivery and cervical laceration. Cervical edema in pregnancy increases the risk of cervical dystocia and cervical lacerations. However, lacerations with edema are predicted to have a poor wound-healing process. The technique with gauze packing presented in this case may be useful in the protective handling of the uterine cervix during pregnancy.

## Introduction

Pelvic organ prolapse is defined as the partial or total descent of pelvic organs through the vagina due to abnormalities of the supporting tissues. It is classified into cystocele, rectocele, enterocele, and uterine prolapse [1,2]. Although uterine prolapse itself is a relatively common disease, the rate of uterine prolapse during pregnancy is considered a rare condition with an incidence of one case for every 10,000 to 15,000 pregnancies [3]. Uterine prolapse during pregnancy may lead to cervical dystocia and hazardous laceration of the uterine cervix [4]. Owing to its low incidence, peripartum management for protruding engorged cervix is rarely discussed. We report here a case of uterine prolapse in pregnancy, which was successfully managed before delivery.

## Case presentation

**Figure 1:**
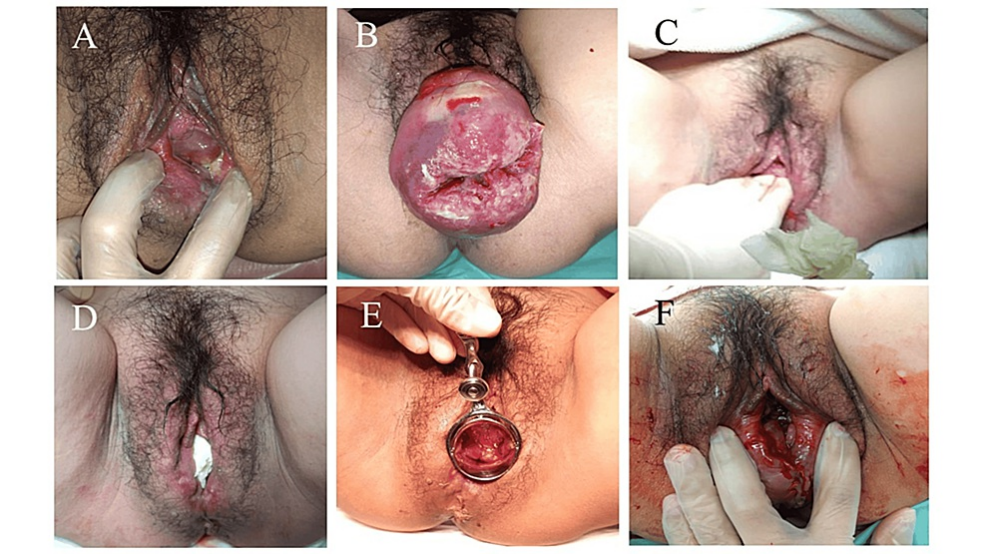
Findings of uterine prolapse before and after delivery Prolapse of uterine cervix at 33 weeks gestation (A) and at 36 weeks gestation (B). Manual retraction of uterine cervix was performed with gaze packing (C, D). Cervical edema was improved the day after the gaze packing (E). No cervical laceration was observed after vaginal delivery (F).

The patient was a 35-year-old multiparous woman (gravidity-2 parity-1), 170 cm tall, weighing 60.6 kg, with a BMI of 20.9. Her history of gestation and delivery was a 3,514 g baby delivered by vacuum extraction three years before. She had conceived spontaneously and was referred to our hospital at 33 weeks gestation due to a descensus of the uterine cervix for 30 weeks gestation (Figure 1A). The most prolapsed point was the Pelvic Organ Prolapse Quantification (POP-Q) score, C: ±0. The patient was then followed on an outpatient basis. At 36 weeks gestation, the prolapse was further exacerbated, so pessary treatment (Milex Support Ring® size 76, Fuji Medical Ltd., India) was started. She had been doing well after the pessary was placed, but at 39 weeks of pregnancy, she was admitted to the hospital because of spontaneous prolapse of the pessary and constant prolapse of the cervix. An image of the prolapsed cervix is shown in (Figure 1B). The most prolapsed point was POP-Q score C: +7 with no cystocele or rectocele. No pain, dysuria, or urinary incontinence was noted, and there was edema of the cervix. We decided that treatment with a pessary would be difficult and decided to pack the uterine cervix with a vaginal gauze due to improve the edema of the cervix. Manual retraction of the edematous cervix was gently performed with gauze packing (Figures 1C, 1D), and strikingly improved edema of the cervix was observed one day after the gauze packing (POP-Q score C: -3, Figure 1E). Furthermore, a traditional Japanese herbal medicine (Goreisan, 10 g/day; Tsumura Ltd., Tokyo) was prescribed for a week to relieve cervical edema. Induction of labor was planned due to the heavy-for-date infant (+2.0 standard deviation, EFW percentile by ultrasound), and the patient uneventfully delivered a healthy male baby weighing 3,970 g at 39 weeks gestation without any cervical laceration (Figure 1F). There was no prolapse of the cervix after delivery. The POP-Q scores on day 3 postpartum were Aa: -1, Ba: -1, C: -4, gh: 4, pb: 3.5, tvl: 10, Ap: -2, Bp: -2, D: -5. She was discharged on the fourth day after delivery, and her POP-Q score at six months postpartum was POP stage 2 (Aa: 0, Ba: 0, C: -5, gh: 4, pb: 3.5, tvl: 10, Ap: -1, Bp: -1, D: -6). She has a mild drooping sensation with no dysuria or urinary incontinence and is being followed up with pelvic floor muscle training without using a pessary.

## Discussion

The rate of uterine prolapse during pregnancy is considered a rare condition with an incidence of one case for every 10,000 to 15,000 pregnancies [3]. This mechanism is thought to be due to increased abdominal pressure from the uterus during pregnancy and weak support of the cardinal ligament and uterosacral ligament [1]. In uterine prolapse during pregnancy, there are two types of cases: those that occur before pregnancy and those that occur during pregnancy. In addition to increased abdominal pressure during pregnancy, physiologic changes regarding softening and stretching of pelvic tissue and lengthening and enlargement of the cervix due to an increase in progesterone and cortisol are involved [1]. Complications of pregnancy with a prolapsed cervix include premature rupture of membrane, premature labor, chorioamnionitis, intrauterine infection, and delivery failure due to cervical dystocia and cervical lacerations [5]. The management of pregnancies complicated by uterine prolapse during pregnancy includes bed rest and Trendelenburg position to reduce pressure in the pelvis [1,5]. The use of a pessary is also considered effective, and it is important to use the most appropriate pessary for each patient, such as a ring type, Gellhorn type, or cube type pessary [5]. There is currently no standard consensus on the method of delivery, and it is thought that it should be individualized according to the degree of uterine prolapse, the weeks of pregnancy, and the wishes of the patient [1,6]. In this present case, we explained the risk of worsening the prolapsed uterus to the patient and decided the vaginal delivery because her cervical prolapse had improved. In the management of vaginal delivery with a prolapsed cervix, Duhrssen's incisions, which are cut at the prolapsed cervix outside the vagina, have been reported as a technique to deal with delivery obstruction and cervical lacerations due to a prolapsed cervix [4,7]. However, reports of elective cesarean sections have increased in recent years, and Brown et al. report that the rate of Duhrssen's incisions has decreased from 52% to 9% in the past 30 years. Cervical elongation caused by cervical edema may be considered when cervical protrusion during pregnancy worsens quickly, as in this instance. Edema of the uterine cervix during pregnancy may worsen if the cervix is left protruding, and it also increases the risk of cervical dystocia and cervical lacerations. However, lacerations with edema are predicted to have a poor wound-healing process [5]. Therefore, we believe that reducing cervical edema prior to delivery is crucial.

## Conclusions

We reported here a case of uterine prolapse during pregnancy, which was successfully managed before delivery. Before labor begins, the top objective may be to vaginally retract the bulging cervix and reduce the edema. Regarding uterine prolapse that worsened rapidly during pregnancy, we think it might be caused by cervical edema. We consider that by moving the cervix back into the pelvis, blood flow to the cervix improves, leading to a reduction in edema. The technique presented in this case may be useful in the protective handling of the uterine cervix during pregnancy.
